# Corps étranger iléo-caecal mimant une maladie de Crohn: rapport de cas

**DOI:** 10.11604/pamj.2018.31.236.16816

**Published:** 2018-12-19

**Authors:** Khaoula El Montacer, Fouad Haddad, Souhaila El Mansouri, Mohammed Tahiri, Wafaa Hliwa, Ahmed Bellabah, Wafaa Badre

**Affiliations:** 1 Service d’Hépato-Gastro-Entérologie, Ibn Rochd, Casablanca, Maroc

**Keywords:** Syndrome de Koening, épaississement iléo-caecal, résection iléo-caecale, granulome sur corps étranger, Koening syndrome, ileo-caecal thickening, ileo-caecal resection, foreign body granuloma

## Abstract

L'ingestion du corps étranger est une situation fréquente en gastro-entérologie, cependant la localisation iléo-caecale reste très rare. L'objectif de ce travail était de rapporter le cas exceptionnel d'un corps étranger iléo-caecal révélé par des syndromes sub-occlusifs. Il s'agit d'un patient âgé de 22 ans sans antécédents pathologiques notables, qui s'est présenté avec un syndrome de Koening évoluant dans un contexte d'altération de l'état général. Le diagnostic d'un épaississement iléo-caecal inflammatoire réactionnel à un corps étranger est posé grâce à l'étude anatomo-pathologique d'une pièce opératoire de résection iléo-caecale après que l'endoscopie, l'histologie des biopsies et l'imagerie scannographique étaient non contributives. En l'absence d'orientation anamnestique, la localisation iléo-caecale d'un corps étranger pose un réel problème de diagnostic différentiel avec les pathologies inflammatoires, infectieuses et tumorales du carrefour iléo-caecal. Elle peut être révélée par des complications à type d'occlusion ou de perforation où l'imagerie tient une place primordiale. L'endoscopie joue toujours un rôle diagnostique et thérapeutique essentiel dans la prise en charge des corps étrangers ingérés limitant ainsi la morbidité chirurgicale quoique celle-ci demeure parfois incontournable et l'étude anatomo-pathologique met en évidence un granulome à corps étranger constitué. Le corps étranger iléo-caecal est une situation rarement rapportée et doit dorénavant être considéré devant toute symptomatologie du carrefour iléo-caecal afin d'éviter au patient les effets secondaires et les complications des traitements lourds.

## Abstract (en)

Foreign-body ingestion is a common cause of admission to the Division of Gastroenterology. However foreign-body lodged in the ileocecal region is very rare. This study aims to report the exceptional case of a patient with a foreign body lodged in the ileocecal region revealed by sub-occlusive syndromes. The study involved a patient aged 22 years with no particular past medical history, presenting with König's syndrome evolving in a context of alteration of the general state. Anatomopathological examination of surgical ileocecal specimen performed after inconclusive endoscopy, histologic examination of the biopsies and scannography, showed ileocecal thickening due to infammatory response to a foreign body. In the absence of anamnestic data, ileocecal foreign body poses a real problem of differential diagnosis including inflammatory, infectious and tumoral diseases of the ileocecal junction. The presence of a foreign body may be revealed by occlusive complications or perforations, hence the essential role of imaging. Endoscopy always plays a central diagnostic and therapeutic role in the management of ingested foreign bodies, thus reducing surgical morbidity (although it is sometimes unavoidable). Anatomopathological study shows foreign body granuloma. Cases of foreign body lodged in the ileocecal region have been rarely reported. Now they should be suspected in patients with any symptom, including ileocecal junction disorders, in order to avoid side effects and complications due to heavy treatments.

## Introduction

L'ingestion du corps étranger est une situation à laquelle les gastro-entérologues sont confrontés régulièrement. L'éventail symptomatique est large. Les localisations au niveau de la valvule iléo-caecale sont rares quoique rapportées. L'imagerie occupe une place primordiale dans le diagnostic. L'endoscopie joue un rôle important sur le plan thérapeutique. Le recours à la chirurgie est de plus en plus restreint, réservé aux rares complications [1]. Notre observation médicale permet de sensibiliser les médecins au diagnostic d'un corps étranger à localisation iléale terminale devant un épaississement du carrefour iléo-caecal même en l'absence de données anamnestiques et radiologiques évocatrices.

## Patient et observation

**Figure 1:**
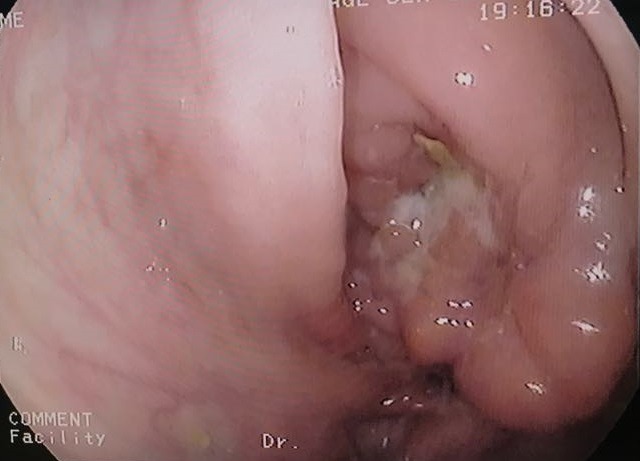
La valvule iléo-caecale infranchissable

**Figure 2:**
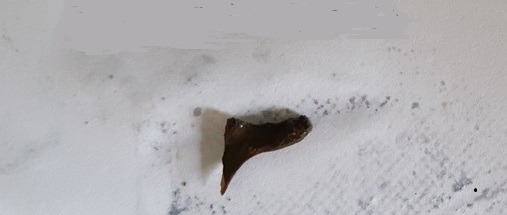
Le corps étranger retrouvé au sein de la sténose inflammatoire

A M âgé de 22 ans, est admis au service de gastro-entérologie du CHU Ibn Rochd pour un syndrome de Koening évoluant depuis 9 mois dans un contexte d'altération de l'état général avec un amaigrissement chiffré à 12kg/8 mois. Aucune notion de diarrhée chronique, ni de contage tuberculeux récent dans l'entourage, ni de néoplasie ou maladies inflammatoires chroniques de l'intestin (MICI) dans la famille. L'examen clinique retrouve un patient apyrétique, stable sur le plan hémodynamique et respiratoire, ayant un IMC (Indice de Masse Corporelle) à 17kg/m^2^, avec un abdomen souple sans masse palpable, ni empâtement, ni épanchement péritonéal décelables. Un syndrome inflammatoire biologique fait d'une anémie hypochrome microcytaire à 11,8g/dl et une vitesse de sédimentation accélérée à 36mm à la première heure est retrouvé. Aucun syndrome de malabsorption biologique n'est noté. Le taux de LDH (Lactate Déshydrogénase) était normal à 113UI/L. La sérologie VIH était négative. L'abdomen sans préparation n'a pas montré de niveaux hydroaériques. Une échographie abdominale a objectivé un épaississement de la région iléo-caecale associé à une infiltration de la graisse péritonéale avec une adénopathie satellite. L'entéroscanner a mis en évidence un épaississement pariétal digestif de la région iléocæcale rehaussé de façon modérée après injection de produit de contraste prédominant au niveau de la dernière anse iléale et du bas fond caecal mesurant 14mm d'épaisseur par endroit. Le patient a eu une colonoscopie à deux reprises montrant une valvule iléo-caecale, oedématiée, pseudopolypoide et infranchissable; le reste de la muqueuse recto-colique était sans anomalies. Les biopsies étagées du colon et de la valvule iléo-caecale ont montré des remaniements inflammatoires chroniques non spécifiques. L'identification du génome bactérien du Bacille de Koch (BK) par méthode PCR (Réaction en Chaîne par Polymérase) est revenue négative. Malgré un test Quantiferon revenu positif (> 10UI/ml) et devant la négativité de la recherche du BK au niveau des expectorations et la normalité de la radiographie pulmonaire, le diagnostic de tuberculose digestive était peu plausible. Le patient a bénéficié d'une résection iléo-caecale avec anastomose iléo-colique termino-latérale. A l'ouverture de la pièce opératoire, on note la présence au niveau de la valvule iléo-caecale d'une lésion blanchâtre sténosante de consistance dure au centre de laquelle un corps étranger ferme, à bords tranchants, remanié, dont la nature n'a pu être déterminée. La paroi abritait un tissu de granulation composé d'un infiltrat inflammatoire fait de lymphocytes, plasmocytes, histiocytes et quelques polynucléaires. Il n'est pas vu de granulome tuberculoïde, ni d'arguments histologiques en faveur de MICI ni de signes de malignité (Figure 1 et Figure 2). Les suites post-opératoires étaient simples. Le patient est asymptomatique, a repris du poids et à un bilan inflammatoire négatif, avec un recul d'un an.

## Discussion

Les corps étrangers (CE) ingérés sont nombreux mais mal répertoriés. Les enfants, les prisonniers, les patients psychotiques ou ayant un retard psychologique et les patients âgés et édentés constituent les populations à risque [1]. Le passage dans le tractus digestif est plus fréquent [2,3]. 80 à 90% des corps étrangers ingérés passent spontanément. 10 à 20% nécessitent des manœuvres non chirurgicales d'extraction quand ils sont accessibles à la voie endoscopique et moins de 1% nécessitent un recours à la chirurgie notamment à la suite des complications (hémorragie, occlusion et perforation) [1,4]. Notre patient est un jeune sans antécédents pathologiques notables ni terrain particulier, présentant un syndrome de koening évoluant dans un contexte d'altération de l'état général. Le diagnostic est habituellement facile et repose sur l'interrogatoire [5], quoique non contributif dans le cas de notre patient rendant l'observation exceptionnelle. Des douleurs rétro-sternales, une odynophagie, une dysphagie, une hyper-sialorrhée et des vomissements peuvent survenir au moment de l'incident qui pourrait également se compliquer de saignement, perforation avec des conséquences infectieuses (médiastinite, cellulite et abcès para-œsophagiens) et fistules du tractus digestif [1,6]. Notre patient a développé un syndrome sub-occlusif sur un processus inflammatoire formé autour du corps étranger au niveau de la région iléo-caecale.

Aucun bilan radiologique n'est nécessaire pour les impactions alimentaires non osseuses sans complications. L'ESGE (European Society of Gastrointestinal Endoscopy) recommande une simple radiographie pour localiser le CE, déterminer sa taille, son aspect et le nombre si l'objet est suspecté être radio-opaque ou dont la nature est méconnue. La tomodensitométrie est indiquée en cas de suspicion de complications pouvant nécessiter un traitement chirurgical [7]. Dans le cas illustré, la radiographie de l'abdomen sans préparation était normale et la tomodensitométrie abdominale n'a montré que la réaction inflammatoire formée autour du corps étranger responsable d'un épaississement au niveau de la région iléo-caecale à l'origine des crises sub-occlusives que le patient exprimait. La nature exacte du corps étranger reste non identifiée, les données anamnestiques n'étaient pas contributives. La recherche d'une maladie sous jacente lors d'un incident d'impaction alimentaire est incontournable et inclue une biopsie du siège de blocage [7]. La pièce opératoire de la résection iléo-caecale que notre patient a subie, était sujette à un examen anatomo-pathologique qui a permis de poser le diagnostic en mettant en évidence le corps étranger au sein d'un granulome inflammatoire tout en éliminant une tuberculose, une maladie de Crohn et une néoplasie. Le granulome à corps étranger est une lésion chronique constituée de manière prépondérante par des cellules de la lignée des monocytes et des macrophages. La fonction de phagocytose y est habituellement bien développée. Il s'y associe en nombre variable d'autres cellules inflammatoires parmi lesquelles des lymphocytes, des plasmocytes, des cellules présentatrices d'antigène ainsi que des polynucléaires neutrophiles et éosinophiles [8]. Dans notre cas, l'infiltrat inflammatoire était constitué de lymphocytes, plasmocytes, histiocytes et quelques polynucléaires. Etait-ce la chronicité qui a modifié le profil cellulaire de cette lésion?

## Conclusion

L'ingestion de CE est une situation fréquemment rencontrée en gastroentérologie. La localisation iléo-caecale est rarement rapportée. Cependant un tel diagnostic doit dorénavant être considéré après avoir écarté les étiologies infectieuses, inflammatoires et tumorales, même en l'absence d'un contexte évocateur, épargnant au patient des préjudices iatrogènes multiples.

## Conflits d’intérêts

Les auteurs ne déclarent aucun conflit d'intérêts.
